# Impact of image-based motion correction on dopamine D3/D2 receptor occupancy—comparison of groupwise and frame-by-frame registration approaches

**DOI:** 10.1186/s40658-015-0117-0

**Published:** 2015-07-29

**Authors:** Jieqing Jiao, Graham E. Searle, Julia A. Schnabel, Roger N. Gunn

**Affiliations:** Department of Engineering Science, Institute of Biomedical Engineering, University of Oxford, Oxford, UK; Imanova Limited, Hammersmith Hospital, 2nd Floor, Burlington Danes Building, London, UK; Department of Medicine, Imperial College London, Du Cane Road, London, W12 0NN UK

**Keywords:** PET, Motion correction, Groupwise registration, Pharmacokinetic modelling, Receptor occupancy studies

## Abstract

**Background:**

Image registration algorithms are frequently used to align the reconstructed brain PET frames to remove subject head motion. However, in occupancy studies, this is a challenging task where competitive binding of a drug can further reduce the available signal for registration. The purpose of this study is to evaluate two kinds of algorithms—a conventional frame-by-frame (FBF) registration and a recently introduced groupwise image registration (GIR), for motion correction of a dopamine D3/D2 receptor occupancy study.

**Methods:**

The FBF method co-registers all the PET frames to a common reference based on normalised mutual information as the spatial similarity. The GIR method incorporates a pharmacokinetic model and conducts motion correction by maximising a likelihood function iteratively on tracer kinetics and subject motion. Data from eight healthy volunteers scanned with [11C]-(+)-PHNO pre- and post-administration of a range of doses of the D3 antagonist GSK618334 were used to compare the motion correction performance.

**Results:**

The groupwise registration achieved improved motion correction results, both by visual inspection of the dynamic PET data and by the reduction of the variability in the outcome measures, and required no additional steps to exclude unsuccessfully realigned PET data for occupancy modelling as compared to frame-by-frame registration. Furthermore, for the groupwise method, the resultant binding potential estimates had reduced variation and bias for individual scans and improved half maximal effective concentration (EC_50_) estimates were obtained for the study as a whole.

**Conclusions:**

These results indicate that the groupwise registration approach can provide improved motion correction of dynamic brain PET data as compared to frame-by-frame registration approaches for receptor occupancy studies.

**Electronic supplementary material:**

The online version of this article (doi:10.1186/s40658-015-0117-0) contains supplementary material, which is available to authorized users.

## Background

Dynamic PET brain scans are susceptible to head motion that distorts the tissue-to-voxel mapping, and this leads to degraded PET images from acquisitions that can last up to 2 h. If uncorrected, motion-induced attenuation correction mismatch, inter-frame misalignment and intra-frame blurring in the PET data will make the quantification of the tracer kinetic data unreliable [1]. Previous approaches to the motion problem have included the use of head restraints or external motion tracking systems that have been developed to try to record the motion parameters [2–5]. However, these methods have limitations either due to patient discomfort, accuracy or ease of use.

Meanwhile, image-based computational methods have been developed to establish the spatial correspondences between PET data at different time frames [6–8], allowing for post-acquisition corrections to be applied. Such methods perform a rigid frame-by-frame (FBF) image registration of PET time frames to a common reference image, which is usually a PET image derived from a single frame, a weighted sum of frames or an associated magnetic resonance (MR) image for the subject. The FBF registration methods have been widely used for motion correction in recent studies [9–12] due to the ease of implementation based on existing publicly available image registration software packages, such as Statistical Parametric Mapping (SPM) (used in [13]), AIR (used in [14]), FLIRT (used in [15]) and the commercial software PMOD. FBF methods are shown to improve the integrity of PET data but have potential limitations that need consideration. For example, the FBF registration is solely based on maximising the spatial similarities between images and it can converge to inaccurate solutions in the presence of noise [16].

Recently, a groupwise image registration (GIR) framework for dynamic PET data has been introduced for motion correction [17, 18]. The GIR method enables noise modelling and accounts for tracer kinetics by incorporating a pharmacokinetic model with either an arterial input function (AIF) [18] or a reference tissue input function [17]. Improved registration results as compared to FBF methods have been demonstrated in simulation-based validations. This work aims to evaluate the motion correction performance of these image-based methods on an occupancy study where the competitive binding can impose further challenges for conducting image registration on the PET images. Data from a dopamine D3/D2 receptor occupancy study with [11C]-(+)-PHNO were used. The study was designed to measure the half maximal effective concentration (EC_50_) of the D3 antagonist GSK618334. For the reconstructed PET time frames, three separate approaches to motion were applied: (1) no motion correction, (2) frame-by-frame motion correction and (3) groupwise image registration motion correction with a reference tissue input. Following motion correction, kinetic analysis was applied to each dataset to derive regional binding potential estimates for each scan and then modelling of the competitive binding of the drug to derive its EC_50_ was performed using all scans in the study.

## Methods

### Human [11C]-(+)-PHNO PET study

The motion correction algorithms were evaluated on [11C]-(+)-PHNO PET occupancy data involving a range of doses of the antagonist GSK618334. Data from eight subjects, from a previously reported study [19], were used here. All subjects were healthy, males, drug-free, non-smoking volunteers, aged between 25 and 55 years and with body weights and BMI in the normal range. All subjects gave written informed consent, and their eligibility was confirmed via medical history, physical examinations and standard tests. Further details of the inclusion and exclusion criteria can be found on www.clinicaltrials.gov by reference to NCT00814957, and the study was approved by East of England Hatfield REC (known as NRES Committee East of England—Welwyn at the time of the study). Each subject received a baseline PET scan, then a single oral dose of 5–550 mg of GSK618334 followed by two further PET scans performed between 1.5 and 29 h post-administration of GSK618334. Venous blood was sampled for measurements of GSK618334 plasma concentration. The [11C]-(+)-PHNO PET scans were acquired using a Siemens Biograph 6 PET-CT with Truepoint gantry in 3D mode and then reconstructed using filtered back projection with corrections for dead time, random coincidences, variations in detector sensitivity, attenuation (based on a low-dose CT acquisition) and scatter. The reconstruction had the measured resolution of 9 mm (transaxial) and 7 mm (axial) in full width at half maximum at the centre of the field of view, and after reconstruction, the PET images were filtered with a Gaussian filter of 5 mm in full width at half maximum in the three orthogonal planes. Dynamic data were acquired using 26 frames (durations 8 × 15 s, 3 × 1 min, 5 × 2 min, 5 × 5 min, 5 × 10 min). Arterial blood data was also acquired and enabled plasma input-based modelling to be applied for determination of the final binding potential BP_ND_ regional outcome measures of interest. Each subject also received a high-resolution T1-weighted MR scan with a Siemens Tim Trio 3T scanner (Siemens Healthcare, Erlangen, Germany). A U-shaped head holder with foam padding designed to snugly hold the subject’s head in place laterally, with a soft Velcro strap across the forehead to aid as a reminder to the subject, was used in this study.

### FBF motion correction

The FBF method co-registers all the PET frames to a common reference based on spatial similarity. The PET frame acquired between 13 and 15 min in the scan was used as the reference frame, and normalised mutual information (NMI) was used as the cost function. The settings of the FBF method used in this work have been optimised in our previous internal investigations. Frames 13–15 min were selected based on previous (unpublished) work which optimised the method by evaluating the performance using a range of possible reference frames for [11C]-(+)-PHNO. The image of [11C]-(+)-PHNO at this time in the scan contains features that are common to both early distribution and later binding phases. Although the duration of this frame (2 min) is short, it balances the desire to select a frame with minimal motion whilst capitalising on the high imaging statistics at this time in conjunction with representation of all the key image features. The FBF algorithm was implemented in MATLAB 7.7, using functions available within the Statistical Parametric Mapping (SPM8, http://www.fil.ion.ucl.ac.uk/spm/) with the default settings for the optimisation, interpolation, image smoothing and histogram smoothing.

### GIR motion correction

The proposed GIR algorithm conducts motion correction by solving the maximum likelihood problem. Assuming the measurement is distributed as a multivariate Gaussian, the likelihood of the measured dynamic PET data **Y** can be formulated as

1 $$ L\left(\varPhi, \mathbf{T};\mathbf{Y}\right)={\displaystyle \prod_{j=1}^M{\displaystyle \prod_{k=1}^F\frac{1}{\sqrt{2\pi {\sigma}^2\left({\mathbf{x}}_j,k\right)}} \exp \left(-\frac{{\left(\mathbf{Y}\left({\mathbf{T}}_k^{-1}\left({\mathbf{x}}_j\right),{t}_k\right)-{\mathbf{Y}}_{\varPhi}\left({\mathbf{x}}_j,{t}_k\right)\right)}^2}{2\pi {\sigma}^2\left({\mathbf{x}}_j,k\right)}\right)}} $$

where **Y**_*Φ*_ is the predicted PET data determined by the tracer kinetic parameter *Φ*, **T**_*k*_ is the spatial transformation that corrupts the voxel-to-tissue mapping for the *k*th frame, *σ*^2^(**x**_*j*_, *k*) is the variance term describing the measurement noise level and *M* and *F* are the numbers of voxels and time frames, respectively.

The unknown **T** and *Φ* can be optimised iteratively until convergence. For brain images, **T** describes the rigid head motion using three translations and three rotations. **Y**_*Φ*_ can be described by the generalised reference tissue model embedded in a basis function framework and solved by the method of basis pursuit denoising [20] as follows.

Let *C*_*T*_(*t*) be the tracer concentration time course in the target tissue, then *C*_*T*_(*t*) can be expressed as an expansion on a basis as ${C}_T(t)={\phi}_0{C}_R(t)+{\displaystyle {\sum}_{i=1}^N{\phi}_i{\psi}_i},$ where ${\psi}_i={e}^{-{\theta}_it}\otimes {C}_R(t)$ and *C*_*R*_(*t*) is the tracer concentration time course in the reference tissue. A discrete set of values can be selected for *θ*_*i*_ from a physiologically plausible range spaced in a logarithmic manner to elicit a suitable coverage of the kinetic spectrum. The measured PET data **Y**(**x**, *t*) corresponds to *C*_*T*_(*t*) as

2 $$ \mathbf{Y}\left({\mathbf{x}}_j,{t}_k\right)=\frac{1}{t_k^e-{t}_k^s}{\displaystyle {\int}_{t_k^s}^{t_k^e}{C}_T(t)dt=}{\mathbf{Y}}_{k,j}, $$

where ${t}_k^s$ and ${t}_k^e$ are the start and end times for the *k*th frame (*k* = 1 ⋯ *F*). Accordingly, the basis functions can be written as

3 $$ \begin{array}{c}\hfill {\varPsi}_{0,k}=\frac{1}{t_k^e-{t}_k^s}{\displaystyle {\int}_{t_k^s}^{t_k^e}{C}_R(t)dt}\hfill \\ {}\hfill {\varPsi}_{i,k}=\frac{1}{t_k^e-{t}_k^s}{\displaystyle {\int}_{t_k^s}^{t_k^e}{e}^{-{\theta}_it}\otimes {C}_R(t)dt},\kern0.5em i=1\cdots N\hfill \end{array} $$

The unknown tracer kinetic parameter matrix of the image *Φ* can then be determined by solving **Y** ≅ *ΨΦ*. In practice, to account for the uncertainty of the measurements, the weighted least squares problem

4 $$ {\mathbf{W}}^{\frac{1}{2}}\mathbf{Y}\cong {\mathbf{W}}^{\frac{1}{2}}\varPsi \varPhi $$

can be considered, where **W** is the inverse of the covariance matrix corresponding to the noise variance term *σ*^2^ in Eq. 1. The noise variance for decay-corrected PET data can be modelled as ${\sigma}^2(k)={\displaystyle \sum_j\mathbf{Y}\left({\mathbf{x}}_j,{t}_k\right)}/\left({t}_k^e-{t}_k^s\right)\times dcf(k),$ derived from the variance model 1 in [21], where $dcf(k)=\lambda \left({t}_k^e-{t}_k^s\right)/\left[ exp\left(-\lambda {t}_k^s\right)- exp\left(-\lambda {t}_k^e\right)\right]$ is the decay correction function and *λ* is the decay constant of the isotope. Given the independence of the frames, **W** is diagonal and can be calculated as **W**_*kk*_ = 1/*σ*^2^(*k*).

The basis is typically overcomplete (*N* > *F* − 1), leading to an under-determined set of equations that basis pursuit denoising solves with the addition of a 1-norm penalty term [20]

5 $$ { \min}_{\varPhi }{\left\Vert {\mathbf{W}}^{1/2}\mathbf{Y}-{\mathbf{W}}^{1/2}\varPsi \varPhi \right\Vert}_2^2+\mu {\left\Vert \varPhi \right\Vert}_1 $$

Here, *μ* > 0 is a regularisation parameter which balances the approximation error and sparseness of *Φ* and imposes a unique solution. To avoid the difference in the scales, the basis functions are normalised here so that ‖*Ψ*_*i*_‖_2_ = 1 for all *i*. We previously proposed an efficient way to determine the value for *μ* [17], and based on this approach, we use a value of *μ* = 8.69 for [11C]-(+)-PHNO.

**Fig. 1:**
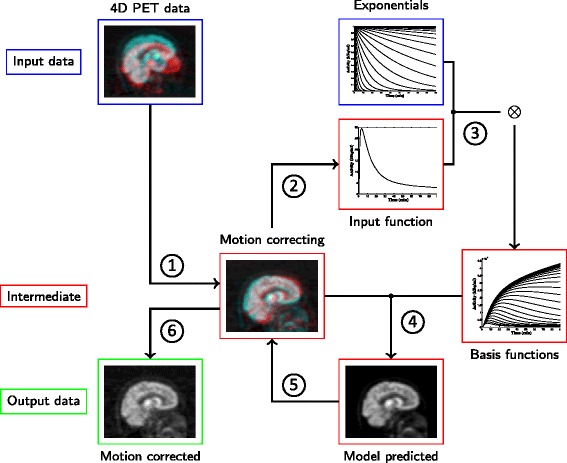
Schematic illustration of the proposed groupwise image registration (GIR) algorithm for motion correction. With the initialisation in step *1*, the algorithm repeats steps *2*–*5* until convergence and returns the final motion corrected dynamic PET data in step *6*

This general reference tissue kinetic model is used as the pharmacokinetic model in the GIR method. It constrains the registration in a groupwise fashion by using the temporal information. The complete algorithm is summarised in Fig. 1. Step 1 initialises the algorithm using the identity function for **T**. In step 2, the discrete reference data is first extracted from the motion-corrected PET data **Y**(**T**^− 1^(**x**), *t*) as a regional time activity curve from the anatomical reference region of choice, and the reference input function *C*_*R*_(*t*) is generated using linear interpolation. For the purpose of describing the tracer kinetics, rather than estimating the absolute parameters for binding or uptake, a region with low specific binding is mathematically appropriate for deriving the reference input. Step 3 calculates the basis functions which are convolutions of the reference tissue input and the pre-defined exponentials. Step 4 solves the kinetic model fitting via basis pursuit denoising. In step 5, the original motion-corrupted PET data **Y**(**x**, *t*) is registered to the model-predicted PET data **Y**_*Φ*_ to update the motion estimation **T** and motion-corrected PET data **Y**(**T**^− 1^(**x**), *t*). Steps 2–5 repeat until convergence, and the algorithm returns the motion-corrected PET data **Y**(**T**^− 1^(**x**), *t*) in step 6.

In this work, for kinetic modelling in the GIR motion framework, the reference input function was derived from the grey matter of the cerebellum for the [11C]-(+)-PHNO PET data. The reference region was delineated via nonlinear registration using SPM8 (http://www.fil.ion.ucl.ac.uk/spm) of a predefined brain atlas [22] to the subject’s MRI (aligned to the PET image) to propagate the segmentation.

### Regional calculation of [11C]-(+)-PHNO BP_ND_

Regional analysis of BP_ND_ and occupancy analysis were performed after (1) no motion correction, (2) FBF motion correction and (3) GIR motion correction with reference tissue input. The [11C]-(+)-PHNO kinetics were analysed for six regions-of-interest (ROIs): substantia nigra (SN), globus pallidus (GP), ventral striatum (VST), dorsal caudate (CD), dorsal putamen (PU) and thalamus (TH). These target ROIs were defined manually according to guidelines described previously [22]. GP, VST, CD, PU and TH were drawn on each subject’s structural T1-weighted magnetic resonance imaging (MRI). The MR T1-weighted image was registered to the time-weighted integral of the dynamic PET images following motion correction using the rigid registration function in SPM8 with a mutual information cost function. SN was defined on each subject’s baseline PET integral image given the insufficient contrast available from MRI data. Regional time-activity curves (TACs) were then derived for each ROI.

Subsequently, a two-tissue compartmental (2TC) plasma input model was applied to the regional time activity curves to appropriately quantify regional [11C]-(+)-PHNO volume of distribution (*V*_*T*_) estimates in the basal ganglia ROIs [19]. This included a fixed blood volume correction of 5 %. Regional ${\mathrm{BP}}_{\mathrm{ND}}^{\mathrm{ROI}}$ estimates were then derived for each of the target regions using the cerebellum as the reference region,

$$ {\mathrm{BP}}_{\mathrm{ND}}^{\mathrm{ROI}}=\frac{V_T^{\mathrm{ROI}}-{V}_T^{\mathrm{CER}}}{V_T^{\mathrm{CER}}} $$

### Competitive binding of drug and PHNO

The [11C]-(+)-PHNO occupancy study was designed to measure the dopamine D3 and D2 receptor occupancy of GSK618334 and requires the application of a two-site competitive binding model [19]. Given the baseline binding potential, ${\mathrm{BP}}_{\mathrm{ND}}^{\mathrm{base}}$, the binding potential following drug administration, ${\mathrm{BP}}_{\mathrm{ND}}^{\mathrm{drug}}$, and the plasma concentration of the drug (GSK618334), ${C}_p^{\mathrm{drug}}$, then,

6 $$ {\mathrm{BP}}_{\mathrm{ND}}^{\mathrm{drug}}={\mathrm{BP}}_{\mathrm{ND}}^{\mathrm{base}}\left(\frac{f_{\mathrm{PHNO}}^{\mathrm{D}3}}{1+\frac{C_p^{\mathrm{drug}}}{{\mathrm{EC}}_{50}^{\mathrm{drug},\mathrm{D}3}}}+\frac{1-{f}_{\mathrm{PHNO}}^{\mathrm{D}3}}{1+\frac{C_p^{\mathrm{drug}}}{{\mathrm{EC}}_{50}^{\mathrm{drug},\mathrm{D}2}}}\right), $$

where ${f}_{\mathrm{PHNO}}^{\mathrm{D}3}$ is the regional fraction of baseline [11C]-(+)-PHNO BP_ND_ corresponding to D3 binding with values of 0.87 for SN, 0.66 for GP, 0.39 for VST, 0.69 for TH, 0.21 for CD and 0.14 for PU [19], ${\mathrm{EC}}_{50}^{\mathrm{drug},\mathrm{D}3}$ and ${\mathrm{EC}}_{50}^{\mathrm{drug},\mathrm{D}2}$ are the plasma concentrations of the drug (GSK618334) that would result in 50 % occupancy of the D3 and D2 receptors, respectively.

Recent work has demonstrated that for true quantification, it is necessary to account for mass effects of [11C]-(+)-PHNO itself and a small displaceable specific signal in the cerebellum in addition to competitive binding of the drug at D3 and D2 sites [19]. For the actual fitting of the [11C]-(+)-PHNO BP_ND_ data, we used an extension of the competitive binding model in Eq. 6 that includes corrections for PHNO mass dose effect at the D3 sites and cerebellum specific binding [19]. This model is given in Additional file 1. Note that when modelling the competitive binding, all regions were fitted simultaneously and the ${\mathrm{EC}}_{50}^{\mathrm{drug},\mathrm{D}3}$ and ${\mathrm{EC}}_{50}^{\mathrm{drug},\mathrm{D}2}$ parameters assumed constant across all regions and subjects [19].

## Results

### Motion correction of [11C]-(+)-PHNO data

**Fig. 2:**
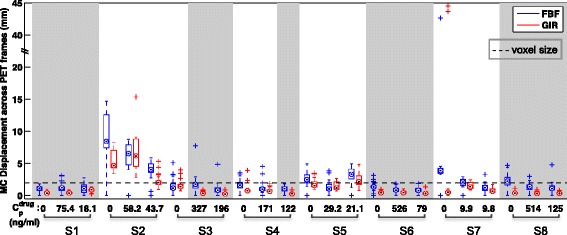
Summary of displacements introduced by FBF and GIR MC methods for [11C]-(+)-PHNO PET occupancy study data. In each *box*, the *central mark* denotes the median, the *edges* of the box are the 25th and 75th percentiles, the *whiskers* extend to the most extreme data points not considered to be outliers, and outliers are plotted individually using the symbol *+*. Subjects (S1–S8) exhibited various degrees of motion during the scans. The scans marked with *grey* background had visually negligible motion following assessment by an observer viewing the data in the movie mode in FSLview. For these scans, the FBF method introduced up to 5 mm displacement, whereas the displacement introduced by the GIR method was at a sub-voxel level. Plasma concentration of GSK618334 is also shown for each scan

The GIR and FBF motion correction algorithms were applied on reconstructed [11C]-(+)-PHNO PET data to address inter-frame misalignment caused by subject motion. PET data that had already been attenuation corrected was used due to the clinical pipeline and the use of PET data without attenuation correction will be considered in the “Discussion” section. Firstly, visual inspection using the movie mode in FSLview (http://fsl.fmrib.ox.ac.uk/fsl/fslview/) was performed on the 24 scans obtained from the eight healthy subjects without motion correction to derive an initial qualitative assessment of motion. In four scans, there was severe motion with up to 10° rotations or 40 mm translations. In eight scans, there was motion at the level of the voxel size (2 mm), and in 12 scans, the motion was difficult to detect. For the motion correction algorithms, a metric summarising the displacement was calculated for each time frame by using the estimated translation and rotation parameters,

7 $$ \mathrm{Displacement}=\sqrt{\frac{1}{M}{{\displaystyle {\sum}_{j=1}^M\left\Vert \mathbf{T}\left({\mathbf{x}}_j\right)-{\mathbf{x}}_j\right\Vert}}^2}, $$

where **T** is the rigid transformation determined by the translations and rotations, *M* is the number of all the voxels and **x**_*j*_ is the coordinate of voxel *j*. Figure 2 shows a summary of the displacements introduced by both FBF and GIR motion correction algorithms for the 24 scans. Whilst the individual reference frames for both the FBF and GIR methods may be different, we are interested in comparing the distributions of the displacements which should be insensitive to this.

The computation time of the GIR motion correction algorithm depended on the amplitude of the motion and the image noise. On a desktop workstation (CPU 3.20 GHz, 16 GB RAM) with MATLAB 7.7, the GIR algorithm took between 20 and 90 min of computation time for each 26-frame dynamic image; the FBF algorithm took in general 60 min. No GPU or parallel computing was applied in this work.

**Fig. 3:**
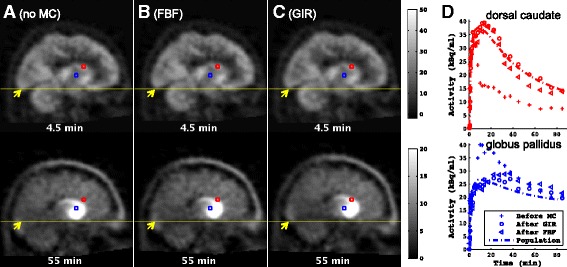
Selected temporal frames from a sagittal slice from subject 2’s baseline [11C]-(+)-PHNO data. Times are mid-frame times. **a** Before motion correction, **b** after motion correction by the conventional FBF algorithm and **c** after motion correction by the proposed GIR algorithm. Units are kBq/ml. **d** TACs from voxels in dorsal caudate and globus pallidus, depicted in colours corresponding to the voxels shown in **a**, **b** and **c**, which are spatially fixed to demonstrate the displacement. The subject exhibited obvious rotation of ~10°, as shown on the sagittal slices in **a**, which was corrected by the proposed method, as shown in **c**. The TACs for these regions were also obtained from all eight healthy subjects’ baseline data after motion correction, and the population TACs were generated by averaging TACs normalised for [11C]-(+)-PHNO dose and subject weight. These population TACs were scaled to match subject 2’s baseline data and are shown in **d**. The tracer kinetics showed consistency with the population data after GIR motion correction. The scan had no GSK618334 taken, and the [11C]-(+)-PHNO injected activity was 384.9 MBq (injected mass 4.24 μg)

**Fig. 4:**
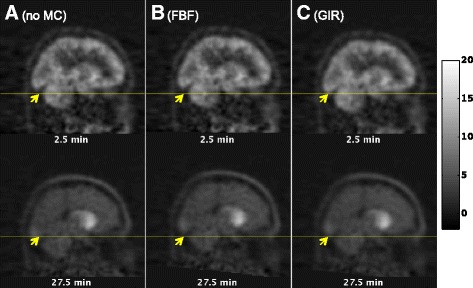
Selected temporal frames from a sagittal slice from subject 2’s follow-up [11C]-(+)-PHNO data. Times are mid-frame times. **a** Before motion correction, **b** after motion correction by the conventional FBF algorithm and **c** after motion correction by the proposed GIR algorithm. Units are kBq/ml. The GSK618334 plasma concentration in the scan was 58.2 ng/ml, and the [11C]-(+)-PHNO injected activity was 139.8 MBq (injected mass 2.35 μg)

Figures 3 and 4 illustrate the performance of the GIR and FBF algorithms when registering [11C]-(+)-PHNO PET data from subjects with visually obvious motion. The motion artefact was well corrected by the GIR algorithm as indicated by the sagittal view of the PET data. Furthermore, for the baseline scan, the voxel-based TACs from dorsal caudate and globus pallidus shown on the sagittal slice are displayed before and after GIR motion correction together with the normalised population TACs for these ROIs. The population TACs were generated by averaging the baseline [11C]-(+)-PHNO PET data after motion correction over the eight healthy subjects and were scaled according to dose and subject weight. The consistency with the normalised population data after MC by the GIR algorithm provides supporting evidence for the successful removal of the inter-frame misalignment caused by motion.

### Binding potential of [11C]-(+)-PHNO

**Fig. 5:**
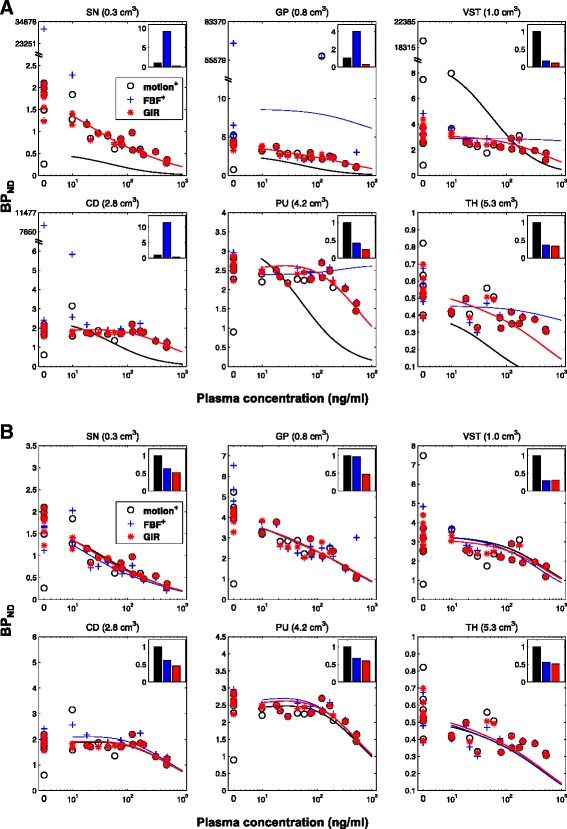
Competitive binding analysis of [11C]-(+)-PHNO data. **a** Fits (shown as *curves*) to the baseline and post-dose data before and after motion correction by FBF and GIR (shown as *circles*, *crosses* and *stars*) using a competitive binding model with unweighted BP_ND_ data. The unweighted sum of squared differences (SSQ) of the competitive model fitting was calculated for each BP_ND_ data set and was then scaled to the SSQ of data before motion correction so that SSQ_motion = 1. Other than for the GIR approach, all methods resulted in some unrealistic estimates of BP_ND_ that affected the fits. **b** Competitive model fits of BP_ND_ data points derived from PET data before MC with removal of unrealistic values; after MC by FBF with the removal of unrealistic values; after only MC by the proposed GIR

Regional estimates of [11C]-(+)-PHNO BP_ND_ were derived for baseline and post-GSK618334 PET scans for SN, GP, VST, CD, PU and TH before and after motion correction. During the 2TC kinetic parameter estimation, two unrealistically large *V*_*T*_ values were obtained for the data with no motion correction, three unrealistically large *V*_*T*_ values were obtained after motion correction by FBF, whereas none were obtained after MC by GIR. For the scans where there were unrealistic values of *V*_*T*_, the motion correction error of FBF was not always visually detectable, suggesting that small residual motion can introduce significant errors into *V*_*T*_ particularly for regions with slower kinetics such as the globus pallidus and ventral striatum. Corresponding BP_ND_ values for baseline and post-dose scans are shown in Fig. 5a with unrealistic values shown above the line breaks. After excluding the unrealistic data points considered as convergence failure, the inter-subject variability was assessed on baseline BP_ND_ by the coefficient of variation (CV)

8 $$ \mathrm{C}\mathrm{V}=\frac{\sigma \left({\mathrm{BP}}_{\mathrm{ND}}\right)}{\mu \left({\mathrm{BP}}_{\mathrm{ND}}\right)} $$

where *σ* and *μ* are the standard deviation and mean across the eight subjects, respectively. The CV values before motion correction, after motion correction by the FBF algorithm and by the proposed GIR algorithm are shown in Table 1, together with the ROI size in cubic centimetres. These data provide further evidence for improved registration with GIR through the significant reduction in the CV of baseline BP_ND_ data across all regions. It was also apparent that the conventional FBF algorithm could lead to subsequent convergence problems in the kinetic fitting, whereas the proposed GIR algorithm avoided such problems, thus eliminating the need to subjectively exclude outliers due to unsuccessful motion correction. The conventional FBF algorithm produced less of a reduction in CV (even after excluding unrealistic data).

**Table 1:** The inter-subject variability (CV) in baseline BP_ND_ before and after motion correction (MC)

Region (ROI size)	SN (0.3 cm^3^)	GP (0.8 cm^3^)	VST (1.0 cm^3^)	CD (2.8 cm^3^)	PU (4.2 cm^3^)	TH (5.3 cm^3^)
CV before MC^a^	0.3699	0.3405	0.6345	0.2825	0.2627	0.5172
CV after FBF^b^	0.1785	0.7411	0.2857	0.1173	0.0848	0.1630
CV after GIR	0.1523	0.0896	0.1822	0.0962	0.0772	0.1606

Unrealistically large *V*_*T*_ values due to unsuccessful convergence in 2TC kinetic parameter estimation as a result of uncorrected motion artefacts were removed. ROI sizes are given in cubic centimetres

^a^Two outlier values removed

^b^Three outlier values removed

### Occupancy and estimation of the EC_50_ of GSK618334

The BP_ND_ of [11C]-(+)-PHNO measured in the follow-up PET data, after dosing with GSK618334, was modelled using the extension of Eq. 6 that is provided in the Additional file 1. The two-site competitive binding model, including correction for PHNO mass on D3 binding and a small specific signal in the cerebellum, was applied to the measured data before and after motion correction. The BP_ND_ values obtained before motion correction, and after applying the FBF and GIR algorithms, are shown in Fig. 5a for each of the six target ROIs, together with the competitive binding model fits. Motion correction using the proposed GIR algorithm avoided the convergence problems that led to data points with unrealistic values, which occurred with uncorrected and FBF corrected PET data.

In practice, to maintain the integrity at the study level, it is possible (though not ideal) to remove the outliers with appropriate testing. Here, we considered BP_ND_ values greater than ten as outliers. In Fig. 5b, the competitive binding model fits are shown using BP_ND_ before motion correction with outliers excluded, BP_ND_ after FBF with outliers excluded, and BP_ND_ estimates obtained directly from the GIR algorithm with no exclusions. Even with all this extra help for the other methods, GIR still produces the best fit to the competitive binding data as judged by its ability to achieve the smallest SSQ.

The primary outcome measures of this study, ${\mathrm{EC}}_{50}^{\mathrm{drug},\mathrm{D}3}$ and ${\mathrm{EC}}_{50}^{\mathrm{drug},\mathrm{D}2}$ of GSK618334, estimated using Eq. 6, are presented along with 95 % confidence intervals in Table 2.

**Table 2:** ${\mathrm{EC}}_{50}^{\mathrm{drug},\mathrm{D}3}$ and ${\mathrm{EC}}_{50}^{\mathrm{drug},\mathrm{D}2}$ for GSK618334 estimated before and after motion correction (MC)

MC Method	Removal of unrealistic BP_ND_ data points	${\mathrm{EC}}_{50}^{\mathrm{drug},\mathrm{D}3}$ of GSK618334 (ng/ml)	${\mathrm{EC}}_{50}^{\mathrm{drug},\mathrm{D}2}$ of GSK618334 (ng/ml)
No MC	Required	15 (−6, 37)	529 (206, 852)
MC by FBF	Required	4 (0, 8)	492 (327, 658)
MC by GIR	Not necessary	10 (5, 15)	539 (419, 660)

Values in parentheses indicate 95 % confidence intervals associated with the parameter estimates

The EC_50_ estimates obtained following GIR are in the range of the values obtained before motion correction with the removal of unrealistic BP_ND_ data points (the ones with significant subject motion) and have smaller confidence intervals.

## Discussion

Image-based registration methods are frequently used in brain PET studies to minimise the impact of subject movement on the outcome measures derived from tracer kinetic analysis. In this work, we have investigated and evaluated the applicability of two such motion correction algorithms for dynamic PET data obtained as part of a clinical dopamine D3/D2 receptor occupancy study with GSK618334.

This involved a more traditional FBF approach along with a novel GIR method that we have recently introduced. The GIR approach incorporates a pharmacokinetic model into the registration process so as to provide additional temporal constraints in the registration process over and above just spatial image similarity maximisation. The input function for the pharmacokinetic model is derived directly from the tomographic PET data using a reference region and therefore the GIR method does not necessarily require any arterial blood sampling. We hypothesised that the application of a spatio-temporal (GIR) method that makes better use of the available data would lead to improved results over the purely spatial (FBF) method.

The performance of the FBF and GIR methods was evaluated using data from a dopamine D3/D2 receptor occupancy study in humans with [11C]-(+)-PHNO. In the PET data, there were different levels of subject motion and a range of signal-to-noise ratios (SNR) due to competitive binding of the drug at varying doses. The performance was assessed directly by visual inspection of the PET data and indirectly by assessing the inter-subject variability in baseline BP_ND_, convergence and residuals of the competitive binding modelling and the drug EC_50_ estimation. In addition to the visually improved removal of subject motion, the GIR method led to more reliable BP_ND_ estimates with reduced variation and bias at baseline and when modelling competitive binding of GSK618334 as compared to the FBF method. It also provided estimates of GSK618334 EC_50_ that were consistent with a previously published study that had employed outlier removal techniques but with reduced confidence intervals [19]. These convergent data all provide evidence that the proposed GIR method yields improved registration for dynamic PET data. On the study level, it increases the statistical power by reducing the motion-introduced variability, and in practice, less PET scans would be required to achieve the same outcome parameter precision once the motion correction is accurately conducted using the GIR method.

The proposed GIR method uses the full dynamic data in addition to spatial similarity, and from a theoretical point of view, it should perform better than the FBF method. For the brain D3/D2 images, whilst there is limited binding data outside the striatum, there is still information available from the delivery and washout to regions just containing free and non-specifically bound tracer. Similarly, for other tracers with different distributions, areas of relatively low signal may still contribute usefully to the motion correction process. The pharmacokinetic model employed by the GIR approach is generic allowing for different compartmental topologies at individual voxels and thus should not only handle the kinetics displayed by a broad range of tracers but even different kinetic behaviour in different regions of the image. Furthermore, it is not necessary for the reference region to be devoid of the target biology, as the data can be quantified subsequently, and a region with the fastest kinetics could be used as the reference region. Thus, the method should be generally applicable to dynamic brain PET data (except perhaps when significant metabolite components contribute to the data). The generalised reference tissue model employed is able to describe a range of different behaviours, which will likely include regions outside the brain. Qualifying how well it is able to describe such data is not strictly necessary in order to assess the performance of the approach (for instance, it would not matter if it did not describe these regions particularly well if the algorithm provides improved performance in image registration over existing approaches). Future studies will explore the utility of the approach with other tracers, and further extension to the deformation motion model could also allow application to dynamic imaging outside the brain as well.

In this paper, we have employed filtered back projection (FBP) for the reconstruction of the dynamic image sequence. We fully acknowledge that the application of iterative reconstruction algorithms could have improved the performance of both the FBF and GIR approaches, but an assessment of this was beyond the scope of this paper. Future studies will evaluate the impact of the reconstruction algorithm in more detail. Our hypotheses are, firstly, that the application of iterative reconstruction algorithms to the FBF method would bring its performance closer to that of the current GIR (FBP) method and, secondly, that the application of iterative reconstruction to the GIR method would further increase its performance.

The reconstructed PET data used in this work represents a very common clinical workflow. In practice, the subject motion introduces mismatched attenuation and scatter correction in the reconstruction, which are theoretically challenging to eliminate with post reconstruction approaches. Addressing these issues, however, requires access to the raw PET emission data and extra fast-processing hardware/software that would impose an undesirable cost for clinical use. The approach proposed in this work is directly based on the reconstructed PET images and in the presence of intra-frame motion artefacts (attenuation, scatter etc.), it demonstrates an improvement in the kinetic analysis of dynamic data compared to alternative image-based methods. Further extension of this approach to fully account for attenuation/scatter mismatch in the PET reconstruction framework will be explored and evaluated in future work.

Besides the image-based motion correction methods discussed in this work, which estimate and eliminate the subject motion using only the measured PET data, subject motion can also be tracked and corrected using additional hardware. However, such motion-tracking systems are not always available in typical clinical settings, and additional processing and calibration are required to ensure the mapping of the motion parameters from the motion-tracking space into the PET image space is accurate. The image-based methods presented provide a more accessible and less demanding way to remove the subject motion in the majority of PET studies.

In summary, we have demonstrated the applicability of a novel groupwise-based imaged registration for improving the quality of data obtained from PET receptor occupancy studies, using only measured PET data. The generic nature of the incorporated pharmacokinetic model means that this should have wide utility across PET neuroimaging studies.

## Conclusions

Groupwise image-based registration of dynamic brain PET data provides an improved method to correct for subject motion. Incorporation of a reference input-based general pharmacokinetic model that requires no arterial blood sampling allows for wide applicability of the technique. The approach has value for increasing the integrity of both individual scan data and outcome measures from clinical studies involving a series of scans enabling increased precision or reduction in the required number of scans.

## Additional file

Additional file 1:
**Two-site competitive binding model with corrections for PHNO mass on D3 binding and specific signal in the cerebellum.**
