# Supplementary material for: Impact of image-based motion correction on dopamine D3/D2 receptor occupancy—comparison of groupwise and frame-by-frame registration approaches
Source: EJNMMI Phys. 2015 Jul 29;2:15. doi: 10.1186/s40658-015-0117-0 (PMC4538721; doi:10.1186/s40658-015-0117-0)
Supplement: Additional file 1: — Two-site competitive binding model with corrections for PHNO mass on D3 binding and specific signal in the cerebellum. [file 40658_2015_117_MOESM1_ESM.pdf]

Supplementary Material

The two-site competitive binding model used in this work including corrections for PHNO mass dose effect at D3 sites and cerebellum specific binding is given by

$$BP_{ND}^{drug} = \frac{BP_{ND}^{base}\left(\dfrac{f_{PHNO}^{D3}}{1+\dfrac{C_p^{drug}}{EC_{50}^{drug,D3}}+\dfrac{D^{PHNO}}{ED_{50}^{PHNO,D3}}}+\dfrac{1-f_{PHNO}^{D3}}{1+\dfrac{C_p^{drug}}{EC_{50}^{drug,D2}}}\right)-BP_{ND}^{base,CER}\left(\dfrac{f_{PHNO}^{D3,CER}}{1+\dfrac{C_p^{drug}}{EC_{50}^{drug,D3}}+\dfrac{D^{PHNO}}{ED_{50}^{PHNO,D3}}}+\dfrac{1-f_{PHNO}^{D3,CER}}{1+\dfrac{C_p^{drug}}{EC_{50}^{drug,D2}}}\right)}{1+BP_{ND}^{base,CER}\left(\dfrac{f_{PHNO}^{D3,CER}}{1+\dfrac{C_p^{drug}}{EC_{50}^{drug,D3}}+\dfrac{D^{PHNO}}{ED_{50}^{drug,D3}}}+\dfrac{1-f_{PHNO}^{D3,CER}}{1+\dfrac{C_p^{drug}}{EC_{50}^{drug,D2}}}\right)}$$

where BP_{ND}^{base} is the true baseline binding potential in the target tissue, BP_{ND}^{drug} is the binding potential following drug administration, C_p^{drug} is the plasma concentration of drug (GSK618334) measured, and D^{PHNO} is the total mass dose of (+)-PHNO (ug/kg) administered. f_{PHNO}^{D3} is the regional fraction of baseline [11C]-(+)-PHNO BP_{ND} corresponding to D3 binding with values of 0.87 for SN, 0.66 for GP, 0.39 for VST, 0.69 for TH, 0.21 for CD, 0.14 for PU, $ED_{50}^{PHNO,D3}$ is the mass dose of (+)-PHNO that results in 50% occupancy of the D3 receptor with the value of 0.04ug/kg.[20] $EC_{50}^{drug,D3}$ and $EC_{50}^{drug,D2}$ are the plasma concentrations of the drug (GSK618334) that would result in 50% occupancy of D3 and D2 receptors, respectively, estimated using the nonlinear least-squares optimiser ‘lsqnonlin’ provided in Matlab R2008b (The MathWorks Inc, Natick, MA, USA).
